# Primary school students’ poetic malaria messages from Jimma zone, Oromia, Ethiopia: a qualitative content analysis

**DOI:** 10.1186/s12889-021-11641-8

**Published:** 2021-09-16

**Authors:** Yohannes Kebede, Abdu Hayder, Kasahun Girma, Fira Abamecha, Guda Alemayehu, Lakew Abebe, Morankar Sudhakar, Zewdie Birhanu

**Affiliations:** 1 Department of Health, Behavior, and Society, Jimma University, Jimma, Oromia Ethiopia; 2 Mizan-Tepi University, College of Public Health, Mizan, Ethiopia; 3 President’s Malaria Initiative, United States Agency for International Development, Addis Ababa, Ethiopia

**Keywords:** Content analysis, Poems, Malaria messages, Schools, Malaria elimination, Jimma-Ethiopia

## Abstract

**Background:**

The engagement of schools in malaria control is an emerging strategy. Little is known about the involvement of students in the development of malaria messages. This study evaluated the message content of primary school students’ malaria poems.

**Methods:**

A qualitative content analysis was conducted to explore malaria messages conveyed in poems produced by students. Twenty poems were purposively selected from twenty schools across rural villages in five districts of the Jimma Zone. Data were analyzed using Atlas.ti version 7.1.4 software. The message contents were quantified in terms of frequency, and including metaphors, presented using central themes, categories, and supportive quotations.

**Results:**

A total of 602 malarial contents were generated, and organized into 21 categories under five central themes. 1) Malaria-related knowledge (causation and modes of transmission, mosquito breeding and biting behavior, signs and symptoms, care for insecticide-treated nets (ITNs), and prevention methods), 2) Perceived threats from malaria, 3)The effectiveness of prevention methods (i.e., related to the adaption of ITNs, environmental cleaning, indoor residual spray (IRS), treatment for fever, and drug adherence practices), 4) Misconceptions, beliefs, and malpractices regarding the cause of malaria and drug use) and 5) Direct calls to the adopt ITN, IRS, clean surroundings, treatment, and drug use. The most commonly conveyed message contents were about the severity of malaria, distinguishable signs and symptoms, calls for community participation for malaria elimination, knowledge of preventive methods, and effectiveness of ITN use. Metaphoric expressions (war and death) were used to convey messages about the severity and the need to manage the prognosis of malaria through the active ITN use, which itself was metaphorically represented as ‘a trap’ to mosquitoes.

**Conclusions:**

The poetic analysis indicated that the students developed and disseminated rich malarial messages, especially on malarial knowledge, and perceptions, beliefs, norms and practices of the local community to prevent and control malaria. Therefore, primary school students can be a source of information and would effectively communicate knowledge, perceptions, and promote malaria related practices, particularly in rural settings.

**Supplementary Information:**

The online version contains supplementary material available at 10.1186/s12889-021-11641-8.

## Background

The magnitudes of malaria morbidity and mortality are still important globally, regionally, and nationally compared to the goals of eradication and elimination [1, 2]. Oromia is the most malaria-prone region with a large number of malaria-related risks and deaths in Ethiopia [3]. Jimma appears to be one of the malaria-endemic zones of Oromia, although a declining trend has been observed [4]. There are several global initiatives and commitments to support efforts to eliminate malaria [5, 6]. Community participation and ownership, communication interventions for social and behavioral changes, and scale-up of workable interventions proven to enhance malaria knowledge, attitude, and practices are some of the strategies supported by most malaria programs globally [3, 7–11].

Social and behavior change communication (SBCC) programs, strategies, and activities are emerging elements of effective global directions to eliminate malaria [9–15]. Existing evidence indicates that approaching SBCC through school is one of the proven effective and efficient means of disseminating information to students and their families. School children and teachers are key agents in communicating and encouraging community-wide malaria prevention and control [3, 6–20]. Also, school is a traditional societal setting crucial for peoples to interact and communicate with and share information among different individuals [21].

School-based SBCC interventions were implemented in the Jimma zone from 2017 to 2019. Different intervention packages were conducted and details of the intervention were reported in a separate article [20]. The involvement of primary school students was one of those packages of the project and they were engaged in developing and disseminating messages to schools and broader communities. In particular, those who were members of health/malaria and mini-media/art clubs were engaged in developing artistic malaria communication materials. They developed malaria messages, commonly, in the form of poems to depict communities’ experiences, perceptions, beliefs, emotions, and express behavioral factors such as knowledge, misconception, self -efficacy, perceived risk /severity and practice related to malaria prevention and control strategies for the target population in a more artistic way and disseminated through mini-media, peer discussions, parent days, community meetings, and school opening or closing occasions [5, 19, 22, 23]. Moreover, the project brought a significant change in comprehensive knowledge, message acceptance, practices of ITN utilization and giving priority to children< 5 years old and pregnant women, environmental cleaning specifically breeding sites for mosquitoes, IRS handling and early treatment-seeking for fever [2]. The project followed the assumption of a socio-cultural model, given that the students develop the malaria messages in the context of their culture and background to reflect communities’ experiences, perceptions, beliefs, emotions and behavioral factors, and disseminate them back to the broader community [24, 25].

However, the content of the messages, including the types of figurative speeches expressed in the poems developed by those students has not been evaluated. Additionally, as per the authors’ knowledge, there is no published work that reported contents of malaria messages used in the poems developed by community members such as students. Therefore, this study explored the content of poetic communication materials developed by primary school students as school-based SBCC interventions for malaria in the Jimma zone by 2017–19. The analysis of poems involves reading the poems, analyzing the lines against the titles, investigating emotions, words and stories in the poems, dominating words, connotative meanings, and interpreting figurative languages (a word or phrase that the meaning is different from the literal interpretation of language) [23–29]. Figurative speech is commonly used while developing and disseminating a message in the form of poems. It is the expression of experiences, beliefs, perceptions, and practices related to malaria control and elimination practices using a certain word or phrase that has a different meaning from the literal interpretation of language or the straightforward use of words [26, 30, 31]. The following are some of the types of key figurative speeches: metaphors, asserts the identity, without a connective such as “like” or a verb as “appears”, of literally incompatible terms; similes, an unrealistic comparison is made, using like or as; personification, assigning of human characteristics to non-humans; synecdoche, is the whole is replaced by the part or the part by the whole; irony, is a statement or a situation meaning is contradicted by the presentation of the idea; metonymy, something is named that replaces something closely related to it; and paradox is a statement that seems self-contradictory but may contain insight into the life [26–29, 32, 33].

The contents were explored through a qualitative content analysis approach; a research method that explores the content or information and symbols contained in written documents or other communication media (in this case poems) and explores linguistic expressions and affective, cognitive, social, cultural, and historical significance [34–36]. Exploring and analyzing the message contents of malaria poems developed and communicated by primary school students has multiple purposes. These are: to understand the contents of messages conveyed through the poems, collect experiences of communicating in contexts, i.e., local mental models and beliefs, evaluate and adapt lessons about the students’ roles in malaria communication, and empower schools and students in successful message development for public health programs through Sociocultural model [24, 25].

## Methods

### Study settings and contexts

The study was conducted in primary schools of twenty rural gandas (i.e., the lowest government administrative bodies) of the Jimma Zone, Oromia regional state, Ethiopia from February 20 and March 5, 2020. Jimma town is located 356 K.M far from Finfinne, the capital of the region in the Southwest direction. The zone area was 50.52 km^2^ and had 21 districts, and 42 urban and 513 rural gandas. There were an estimated number of 3.2 million people, with the majority of the population living in rural areas [37]. The study schools were centers of execution for a two-year school-based malaria project aimed to advance community practices from 2017 to 2019 and in five selected higher malaria burden districts of the zone: Limmu-Kossa, Botor-Tolay, Gera, Shebe-Sombo, and Nono-Benja. In these schools, the project engaged students as malaria messengers and artistic message developers for in-school, community, and opportunistic dissemination.

### Study design

A qualitative content analysis was conducted to analyze malaria poems developed and disseminated by primary school students. It was used to analyze text data, and messages of communication materials to provide knowledge and understanding of the contents and figurative speeches used in the poems or phenomena under the study [36, 38–41]. There are three approaches to qualitative content analysis: conventional, directed, and summative. All approaches used to interpret meaning from the content of the text data. The major difference is the way of coding schemes, origins of codes, and threats to trustworthiness [39]. However, this study employed a conventional content analysis approach and the codes, categories and central themes were derived inductively from the data. First, the existing concepts are identified and quantified. Subsequently, the contents were interpreted.

### Population and sampling

Over the school-based malaria SBCC project periods (2017–2019), students developed poems in all twenty study schools that were centers of execution for the project to advance community practices. These schools were selected considering the high number of students enrolled in the school (range, 440–1450 students), located at higher malaria burden areas (previous year’s annual parasitic incidence ranged from 2.1 to 14.1), feasibly accessed based on distance from the district capital (range, 22–75 km), and active engagement and better monthly poem production performance of anti-malarial clubs (based on monthly reports, more than five poems reported as good performance). All poems produced in these schools were the parent population. Table 1 presents the distribution of poems considered in the analysis. Initially, eighty poems were selected across the study schools from a total of 657 poems. Table 1 indicated procedures for selecting poems. A criterion sampling technique was used to select poems from school documentation based on the richness in content, minimum of a page length, readability, and representations by villages, schools, grades, and gender of the poet students. Poems that were unreadable, very short, and shallow in terms of content were excluded. Finally, twenty poems were considered for the content analysis. The final number of poems analyzed was decided based on the saturation of ideas upon agreement among the investigators. A minimum representation was given to the districts, villages, schools, grades, and gender to maximize data triangulation.

**Table 1:** Sampling distribution of the selected poems, schools in rural villages of Jimma zone, Oromia 2020

Districts	Study villages where schools are located (number of poems-sex)				Sample	Sex of poet
Shebe-Sembo	M/sedecha (1F)	Y/dogena (1F)	Mirgano (1 M)	Kishe (1 M)	4	M(2),F(2)
Limmu-Kosa	Ambuye (1F)	Gumar (1 M)	D/Gebana (1F)	C/Ifeta (1 M)	4	M(2),F(2)
Gera	K/Kindibit (1 M)	Sedi (1 M)	G/Challa (1F)	Dusta (1F)	4	M(2),F(2)
NonoBenja	Illu (1 M)	Ebicha (1 M)	Amido (1 M)	Kolatie (1F)	4	M(2),F(2)
Botor-Tolay	B/Adare (1F)	L/Botor (1F)	B/Barite (1 M)	K/Boso (1 M)	4	M(2),F(2)
^a^Grade(poem)	grade 5 (5)	Grade 6 (5)	Grade 7 (5)	Grade 8 (5)	20	M(10),F(10)

^a^Equal poems were allocated to the grades and sex of the poetic students. *F* Female, *M* Male

### Data sources and collection methods

Five experienced individuals (three masters of public health students and two graduated bachelors) were employed to collect poems from schools per district. They received training on the study, sampling criteria and poem selection. Data collectors approached the sites, secured permissions from school focal persons, reviewed documentation, and selected an extra number of information-rich poems. Later, the investigators chose the best poems based on readings according to the representations stated in Table 1.

### Data analysis

A conventional (inductive) content analysis approach was employed to analyze the data. This means that the contents, codes, categories and themes were generated from the text data [36]. First, the selected poems were converted word by word to soft copies and saved in a word document. Texts with plain and straightforward meanings were translated and coded in English. The data analyses were managed using ATLAS.ti 7.1.4 software. Coding was performed by reading and re-reading the compiled poems. Before the actual coding began, three master-level investigators independently read the poems to identify key themes and develop a code structure. To enhance dependability, the coders independently applied the codebook to six selected and rich poems and reviewed for any differences in their coding, which were discussed and resolved. The code structure later evolved as the actual coding progressed by the lead coder. Thus, peer debriefings were conducted among the initial coders’ teams during the coding process. The final code version referred in (Supplementary file 1) was approved by two-PhD holders and one MPH holder experienced researchers. Then, the lead coder analyzed the whole poem using the established structure. Texts in figurative languages were carefully interpreted as equivalent meanings based on the definitions of key types of figurative speech. The interpretations were audited by language and literature experts for accuracy. As the analysis unfolds, potential categories and themes were generated by clustering codes and categories, respectively. Networks across themes and categories referred in (Supplementary file 2). Next, definitions for each category and code were developed. Tick description was provided to the themes. Finally, the results were presented using the major themes, categories, and supportive quotations. Moreover, counts of contents were tabulated to display frequencies of their occurrence across the poems.

### Trustworthiness

The rigor of this study was ensured through credibility, dependability, transferability, and confirmability [36]. The credibility of this study was ensured by involving experienced teams in qualitative research, and coders to develop a code book that guided the data analysis. Tick descriptions were provided to the themes and categories of message content. The findings were presented with supportive quotations which add value to the credibility of the contexts of interpretations. The investigators were experienced qualitative researchers, public health and malaria experts. They intended only to answer the research question of the study that is how effectively students can develop poems that are rich in malaria messages. Consequently, as much as possible, the investigators reported the actual meanings of the contents in the poems with minimal interpretation bias. To ensure this, they maintained subjective neutrality and bracketed themselves, not to intentionally provide expertise meanings than the consistent interpretations transpiring during the coding process. Peer debriefing and daily interactions among each other maintained the credibility of the findings. The diversity of districts, schools, grades, and gender representation in the selected poems can increase the transferability of the findings. Additionally, a saturation of the findings can be witnessed by discussions on malaria communication programs. Finally, the adequacy of the findings in revealing malaria message contents located in students’ poems can be confirmed by the readers based on audits of internal evidence for the integrity of the entire process, and external evidence about malaria communication programs.

## Results

### Profiles of poetic students and poems

**Fig. 1:**
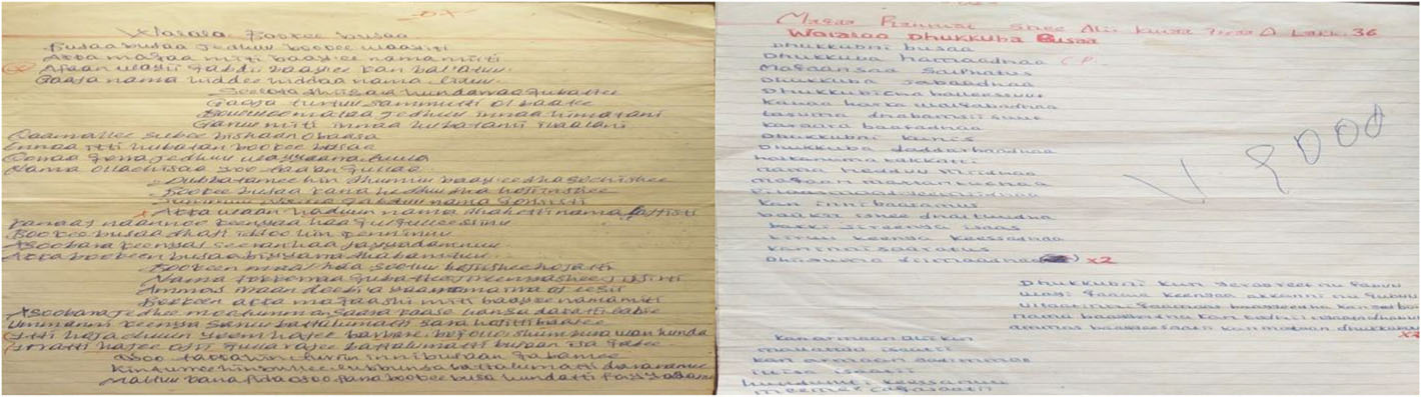
Photos of two selected poems, March 2020, Jimma, Ethiopia (the image depicted in Fig. 1 captured by investigators)

In this study, twenty malaria poems were analyzed to understand the messages conveyed. The ages of students who developed the poems ranged from 12 to17 years. Figure 1 presents photos of the selected poems.

### Message contents of malaria poems

A total of 602 specific contents were generated from poems and organized into twenty-one categories. These categories were organized into five themes: Knowledge about malaria and its prevention; perceptions of threat from malaria; effectiveness of preventive measures; misconceptions, beliefs, and malpractices; and manifest calls to adopt malaria control and preventive behaviors and social changes. Moreover, the central themes of message contents were mainly expressed by metaphoric models. The identified specific codes were described for meaning and followed by detailed descriptions of the central themes and categories.

### Quantifying the message contents of the poems

Table 2 presents details of the frequency of occurrences for themes and categories of message content across the poems. To assess the intensity of the underlying messages and the extent to which they were conveyed, the identified message contents were quantified in a frequency table considering a total count of 602 codes as a denominator. The commonly conveyed contents were messages about the severity of malaria (101), distinguishable signs and symptoms (66), calls to the practice of malaria prevention and elimination (63), and effectiveness of ITN use (49). Given that the hidden meaning of figurative speech identified in this study is considered in a separate article, the details of the counts were not exhaustively presented in the current report. Nonetheless, count analysis of occurrence limited to three selected speeches: Metaphors (27), personifications (18), and similes (7); indicating a total of 52 figurative speech counts.

**Table 2:** Frequencies of themes and sub-themes of malaria messages contents in twenty primary students’ poems Jimma zone, Oromia, Ethiopia, March 2020

Key malaria messages and categories, *n* = 602	Counts
Thematic message 1: Knowledge about malaria	216
Signs and symptoms	66
Ways of prevention and control	59
Causations and mode of transmission of malaria	51
Caring for nets	17
Mosquito breeding behavior	13
Mosquito biting behavior	10
Thematic message 2: Calls to adopt practices, and changes	161
Social changes towards malaria elimination	63
Clean the surrounding environment	42
Utilization of ITN	23
Take precautionary measures for IRS	18
Seek treatment and drug use for malaria	15
Thematic message 3: Threat perceptions from malaria	123
Perceived severity	101
Risk conditions	15
Perceived risk or vulnerability	7
Thematic message 4: Perceived effectiveness of measures	76
Insecticide treated nets/nets (ITNs)	49
Cleaning surrounding	12
Use of drugs	6
Treatment of malaria or suggestive symptoms:	5
Indoor residual spray (IRS)	4
Thematic message 5: Misconceptions, beliefs, and malpractices	26
Preventive and treatment malpractices	23
Misconceptions about causes	3
^a^Total message occurrences	602

^a^Counts of figurative speech were excluded from the total and independently reported in the text

### Thematic message 1: knowledge about malaria

Knowledge about malaria and its prevention was a profound contents conveyed by the poems. The main poetic message contents were about the causes and modes of malaria transmission, mosquito breeding and biting behaviors, distinguishable signs and symptoms, and ways of prevention, treatment, and control of malaria.

#### Causations and mode of malaria transmission

The poems profoundly stressed the cause of malaria. The poetic messages specified that malaria is caused by a parasite plasmodium and transmitted through the bite of a female anopheles mosquito which feeds on human blood. The poems also conveyed a message that the bite of female anopheles mosquitoes causes malaria only when a previous bite is made with someone who has the parasite plasmodium in the bloodstream.

For example, a grade 5 female student from the Botor-Tolay district indicated in her poem:

*“…All mosquitoes do not cause the disease malaria*

*It is caused by a female mosquito that feeds on red blood cells*

*It is called an anopheles mosquito*

*While the parasite is known as Plasmodium...”*

Another grade 5 male student from the Gera district wrote:

*“…Malaria is transmitted by the female anopheles mosquito*

*The causing parasite is called Plasmodium.*

*The mosquito transmits the diseases to another person*

*By first sucking blood, this contains Plasmodium parasite*

*It further transmits the diseases to other people.”*

The poems noted that a single mosquito can reach many people within a day or an entire community within a short period of time to transfer malaria. By this content, the students conveyed the latent message of potential risks that the mosquitoes can cause if preventive measures are not consistently used in all seasons irrespective of low or reducing malaria infection.

*“…Anopheles mosquito bites a lot of people in one night*

*Not the bites alone, it the disease that is transmitting...” (Grade 7 student, Nono Benja).*

#### Mosquito biting behavior

Interestingly, the students also conveyed the biting behavior of the mosquitoes, that is, it bites mainly during night time to suck blood to obtain elements that are required for reproduction. For example, a 8th grade female student from the Gera district said the following in the local language

*“…Afaan wayii qabdi baayyee kan bal’atuu*

*Gaafa nama hidde hidda namaa lixuu*

*Kan nama cininitus walhormaataaf dha*

*Yeroon inni nama cininuus sa’ati galgalaa dha…”*

Its literal definition is that the mosquito has a very sharp mouth. When it bites its sharp edge directly enters the bloodstream. The reason for the bite is mainly for the sake of reproduction. And the biting time is often in the evening and night.

The students’ poetic messages specified that the mosquito breeding sites are stagnant water, broken materials that hold water, areas with abundant grass, and swampy areas. It was also mentioned that most of the time, mosquito breeds during winter and summer.

For example, a grade 5 female student from the Botor-Tolay district said:

*“…the mosquito breeds in broken materials that hold water,*

*Multiply in swampy and abundant in the grass*

*Often proliferate during winter, but also in summer*

*It breeds on the accumulated and stagnant water*

*And lays its eggs, 50-70 at one time*

*Completes its life cycle of metamorphosis*

*Then there will be plenty of them all over the world...”*

#### Signs and symptoms

In their poems, the students included signs and symptoms of malaria, such as fever, headache, chills, feeling cold, weakness, joint pain, relapse and vomiting while developing and disseminating the messages. For example, a grade 8 male student from the Botor-Tolay said the following:

*“…These are the symptoms of malaria:*

*It warms the body and causes a headache,*

*Makes you vomit and hate food to eat,*

*It weakens the body, though you may get better,*

*It is on and off as it comes again and again,*

*It is worsening in infants and pregnant women*

*Causing to feel cold it shakes the body,*

*Finally, feel fatigued and lose your consciousness…”*

Interestingly, the students noted a message content that these symptoms might relapse if they were not appropriately treated or took the drug completely. For example, one grade 5 female student from the Shebe-Sombo district said the following in the local language:

*“..Har’aaf nuudhiiftuus hoo isiino boor kaati*

*Dawaa namaa hiruun baayyee dhorkaasaati*

*Yoo fuudhannee fixne maalumanuu gooti (read 2X).”*

Its literal interpretation is that if we interrupt taking the drugs, malaria would relapse. It is strictly forbidden to share drugs with anyone or interrupt. What can malaria do if we take the drugs completely? Therefore, the students emphasized in their poems that to prevent malaria, the community should have to complete the prescribed drugs and it is forbidden to share the drugs with other people.

#### Methods of malaria prevention and control

The students profoundly presented in their poems the methods of prevention, treatment, and control of malaria. Active use of ITN, IRS, and cleaning environment through discharging stagnant water and avoiding broken materials that hold water were mainly mentioned preventive methods. They also mentioned those pregnant women and under-five children should be given priority if there is inadequate ITN. In a local language, a grade 7 male student from Nono-Benja said:

*“…Karaan ittisa dhibee kanaa*

*Karaa heeddu danuusatu jira*

*Bakka hormaata bookee busaa sirritti qulqullessu*

*Mana fincaani qadaadu, qodaa cabaa bishaan kusuu gatuun ballessu*

*Boolla bishaan kuusan keessaa yaasuun gogsuu*

*Raqa waanxuraawaa naannoo irraa fageessu*

*Saaphanasiree fannisuu*

*Yoo haanqinni saaphanaa jiraate dubartii ulfaaf dursa laachu*

*Daa’imman umuri shanii gadiif fannisuu*

*Faayidaa biraaaf akka hinoolle ummata barsiisuu*

*Farra bookee busaa manatti biifsisuu.”*

In this stanza, the student indicated cleaning the surroundings, sleeping under ITN (including giving priority to pregnant women and under-five children when there is a deficit), and IRS as methods for malaria prevention and control.

The students also indicated that malaria is best treated at health facilities including community-based health posts. It was also mentioned that adherence to prescribed drugs is important for improvement. For instance, a grade 5 male student from Gera said:

*“…When we learn that the symptoms suggest malaria*

*We need to go to a health facility within one day*

*Understand and correctly practice the advice of health providers*

*Soon will manage it and will be free from the diseases…”*

#### Care for ITN

In the poems, messages about ITN care were indicated. They mentioned that ITN needs regular washing, drying under a shadow, stitching when torn, and never used for any other purpose. A grade 8 male student from the Nono-Benja district said:

*“…If bed net becomes dirty, wash and dry it under the shade.*

*If it becomes torn, repair or mends it.*

*When it gets 6 O’clock, suspend it carefully*

*Use it wisely and do not make it for other purposes…”*

### Thematic message 2: misconceptions, beliefs, and malpractices presiding in the community

In their poems, the students presented some misconceptions and beliefs related to the community. The beliefs mainly focused on the nature, causes, and prevention of malaria. Perhaps, these could be raised related to the knowledge contents mentioned earlier.

#### Misconceptions about the causes of malaria

Regarding the cause of malaria, the students revealed that their community beliefs about eating sugarcane, dirty foods, hungry, and cold weather cause malaria. Literally, one stanza from a poem developed by a 7th grade female student from the Botor-Tolay said:

*“…People talk different things about the cause of malaria*

*The cause of malaria is not what you think:*

*It is not a lack/shortage of food,*

*It is not sleeping outdoors during day time.*

*If you ask me what is the right cause?*

*I will tell you there is only one, and one right cause*

*The name of transmitting mosquito is the anopheles*

*While the bacteria are called plasmodium…”*

#### Preventive and treatment malpractices

The poems picked that people shared some unhealthy norms regarding preventive/treatment of malaria. They did not seek treatment early when signs of malaria are recognized, use previously saved drugs, share the drugs with others, and use ITNs as fastening rope, sacks, etc. Regarding treatment-related malpractices, one grade 6 male from Gera stated:

*“Qoricha waliif hiruun haalamaaf nu taati?*

*Qarshii keenya baasuun fayyuudhaafi miti?*

*Qooricha fuudhatani nama biraaf laachuutu mul’ata*

*Mana yaalaa deemnaan qoricha ni argannaa*

*Qoricha argannes baasne gatuu miti*

*Sirnaan fayyadamaa akka dawaa ta’utti.”*

Literally, this stanza is asking people about why they make habit of saving or sharing or interrupting drugs and do not seek care as early as possible, and which finally advised appropriate use of drugs.

Regarding misuse of ITNs, a grade 6 male student from Botor-Tolay wrote in the local language:

*“Agoobara jedhe motummaan gaaraa kaasee hanga dakatti raabse*

*Ummanni keenya garuu battalummatti gara hojitti baase*

*Itti hojjechuun yoom hafe barbarree, boqollo,shumbura waan hunda irrratti afe*

*Agobara argannee gaafa ji’a lamaa*

*Funyoosaatu fo’ama jabbootaan hidhamaa*

*Waa’ee fayyaa dhifnee horiin fayyadamna*

*Kun meesirri miti maaluumaaf gowwomna*

*Fayyaa keeyna dagannee maaliif horiin hidhanne?”*

These lines explicitly talked about the government’s commitment to mass distribution of ITN versus people’s misuse as rope and sacks. It also indicated that is foolish to misuse ITN that is intended for people’s health. Finally, urged people for how long they would continue to be reluctant of their health while using ITN for unintended purposes.

### Thematic message 3: threat perceptions from malaria and risk conditions

In the context of this study, the theme of the perceived threat refers to content that were included in the poems regarding risk conditions, perceived risk, and perceived severity.

#### Perceived risk or vulnerability

Across the poems, students were initiating the community to feel the risk of malaria infection or its serious form. It seems that in the community people started ignoring the risk of attack by malaria*.* To illustrate this, 5th grade male student from the Limmu-Kossa stated:

*“…Malaria attacks all persons:*

*Without permission and difference in age*

*Children and aged people*

*Everyone is potentially affected by it*

*It doesn’t fear the fatty ones*

*And never undermine the thin*

*Everyone should keep…”*

The students indicated pregnant women and under-five children are at high risk of experiencing a worsening form of malaria and needing priority for sleeping under ITN. A grade 6 female student, Nono-Benja said:

“*…Giving priority for infants and pregnant is mandatory*

*We don't mean malaria doesn’t infect others*

*But it mainly hurts/affects them…*

*To save ourselves from malaria disease*

*We sleep under the bed net, especially the children and pregnant*

*They are more likely to get malaria;*

*This is because their bodies are weak and vulnerable.”*

In the meantime, the poems warned the community to actively watch over their health from the risk of sudden potential attacks by malaria and not ignoring seeking care anytime they recognized symptoms. A female student in grade 5 from Botor-Tolay mentioned:

*“…Dhukkubichaan yoo qabamne*

*Mana marfee deemu yoo baannee*

*Sa’ati 24 carraan nuuflaatame*

*Kana hunda goonaan carraan*

*Fayyuu keenya yeroohidhoo keessatti*

*Kana itti amanaa hundumti keessani”.*

The lines urged the community to feel as they are at risk of malaria infection, check their health regularly, and early seeking care within the 24-h onset of symptoms.

#### Risk conditions

The students mentioned that the presence of wastes, grasses, plants with flat leaves, swampy areas, stagnant waters, and broken utensils are favorable conditions for the mosquito to breed. So long as people live in such an environment, they are at risk of malaria.

*“…There are accumulated water in our surrounding*

*Where the mosquitoes breed and multiply the disease.*

*They come to bite us and give us the disease.*

*So long as people don’t dry accumulated water in our area”(Grade 5 male, Limmu-Kossa)*

*“...if we don’t clean our environment*

*if we don’t manage to safely dispose of dirty water*

*Mosquito breeds there, and transmits the disease” (grade 8 male student, Shebe-Sombo).*

#### Perceived severity

The poems memorized several stories about the seriousness of malaria that happened in the community over the last few years. The death stories it caused and its effects on farming activities were the main examples of the explanations given to the seriousness of malaria*.* Illustratively, 8th grader male student from Gera stated in local language:

*“…Haaxxummaa busaa mee hubadhaa*

*Lola baayyee ulfaataa dha*

*Otuma hin beekin hubde nama fixxi*

*Utuma hin yaadiin lubbu namaa baasti*

*Maalii kan busaati baayyee jabaataa dha*

*Malli busaa baayyee ulfaataa dha*

*Dhaloota fixate abboota hin jenne*

*Hundarraa qubatte dhaloota hunda fixxee*

*Lafa mancaafte(read 2X) yommuu ilaalan*

*Ariifachisuun lubbu namaa baasaa*

*Jireenya hanqisa alafa irraa ballessaa (read 2X)*

*Duri hundaa jjeese*

*Omishaa fi omishtumma gad xiqqeesse*

*Lammi kan koo beelatti hanbise.”*

This portion literary explained that malaria has taken the lives of many people over the past few years, caused sudden deaths, almost killed someone from every household, and affected farming, and contributed to hunger.

### Thematic message 4: perceived effectiveness of preventive measures

Introductory knowledge contents about key malaria control measures were reported previously. The current theme elaborated how the poems presented the effectiveness of the measures in putting malaria under the control of the community.

#### Insecticide-treated nets

The students stressed in their poems that active use of ITN (any net) can safeguard a family and community from the risk of malaria. ITN can trap malaria-causing mosquitoes. It was presented as a frontline preventive material in the fights against malaria. An 8th grader male student from Botor-Tolay said:

*“…Bed net is a treatment*

*It has a chemical that burns malaria.*

*By it, you can avoid mosquito contact with your bloodstream.*

*You can protect yourself from malaria by sleeping under the net.”*

#### Indoor residual spray (IRS)

It was sketched as an outreach activity to attack mosquitoes that cause malaria. It was believed to effectively kill mosquitoes while resting indoor and during peak breeding seasons.

*“..It is possible to eliminate malaria mosquitoes*

*By getting antimalarial chemical spray in the home.”(Grade 6 male student, Botor-Tolay)*

#### Cleaning surrounding

Regular cleaning of the compounds and surrounding environments was perceived as an effective means of reducing risk conditions or chances of mosquito breeding. An 8th grader from Limmu-Kossa stated in the local language:

*“…Naannoo jireenya keenya saatitti haa ilaalamuu*

*Lola cisaa yaafne yeroo yeroon ilaalle haa qulqullefamu*

*Naannoo jireenya keenya yeroo yeroon ilaalla*

*Naannoo jireenya koo irraa sifageessa*

*Naannoo qulqulessine of irraa eegannaa.”*

This literally means that regular cleaning of the surrounding is an effective way of protecting their locality from mosquitoes and malaria. The poet committed oneself and others to engage in cleaning and get protected.

#### Treatment of malaria or suggestive symptoms

The poems promoted the nearest health facility to effectively test, ruling-out, and manage malaria. A grade 6 male from Gera said the following:

*“…If you are infected with malaria,*

*Go to the nearest health facility*

*Where you can get a treatment*

*If we recognize we have malaria symptoms*

*We should not die from sleeping in bed*

*Immediately get life-saving treatment, which is our duty.”*

#### Proper use of drugs

The poems are contented with considerable messages about malaria drugs. The relapse of malaria disease was presented as a result of failure to complete taking anti-malaria drugs. Professional prescriptions are the only best means of getting proper and quality drugs.

A grade 5 female student from Botor-Tolay stated:

*“If you observe anyone with symptoms of malaria*

*Take the person to a nearby health facility*

*Why are you eaten by malaria while knowing this fact?*

*Completing the drugs given by the health provider is a must*

*Leaving the drugs unfinished is our fault*

*If the drugs are completely taken, then malaria disappears from our body.”*

A grade 8 male from Nono-Benja wrote:

*“…When we see symptoms of malaria,*

*Visiting health facility within 24 hours*

*Following the health professional’s advice correctly*

*Taking the drugs as prescribed by the health professional*

*Refraining ourselves from giving the drugs to someone else,*

*We can be saved from malaria.”*

### Thematic message 5: calls to adopt the control practices, and social and behavior changes

Closely linked to knowledge contents mentioned earlier, there were direct calls across the poems to adopt malaria control measures. Mainly the calls were focused on practice activities related to cleaning the surrounding environment, active use of ITNs, caring for IRS, seeking treatment for recognized symptoms, and appropriate drug use. Interestingly, some contents called for social change and collective engagement in eliminating malaria from their community and beyond.

#### Clean the surrounding environment

Across the poems, the central messages left to the community regarding malaria via the act of cleaning the environment were multiple and fundamental. In the first place, the poems conveyed to the community to have an internal locus of controlling malaria in that they can manage the breeding site or attack by mosquitoes. A student of grade 6 from Botor-Tolay said:

*“…We do not give place to malaria*

*Let's clean up our environment*

*Let’s remove the mosquito’s breeding site*

*So that put our enemy under the control.”*

Secondly, to achieve control over malaria, the community should manage small water bodies/swampy or conditions that are suitable for mosquito breeding. An 8th grader from Limmu-Kossa indicated:

*“..Qodaa caccabelle boollatti awwaalamaa*

*Balfi garaa garaas diidatti gubamaa*

*Bishaan ciises yaa’ee gadhiifamaa*

*Lafa margaa kessatti baay’inaan argamaa*

*Lafti margaalleqe’erraa haamamaa*

*Naannoo jireenya keenyaa yeroo yeroo nilaalla.”*

These lines appeal to the community to dispose of broken materials in a pit, burn solid wastes, cut the grasses, release any accumulated and irrelevant waters, and make these entire things regularly.

#### Take precautionary measures for IRS

In support of efforts to kill mosquitoes and control malaria, there were messages in the poems that commanded the community to adopt regarding IRS. One is precautionary measures following the spray, including proper ventilating and not painting the walls within 6 months of spray. A male student of grade 6 from Shebe-Sombo said:

*“...The anti-malarial chemical spray is the third method*

*Let’s get out of the home while spraying.*

*We will not get back home up until two hours*

*Never open the doors within 15 minutes of spray…”*

Another student of grade 8 from Nono-Benja stressed,

*“..When an anti-mosquito chemical is sprayed,*

*Refrain yourself from painting and posting*

*Never paint and mold the wall with mud*

*Follow this command up until 6 months*

*The spray has anti-mosquito chemicals.*

*The sprayed drugs work up to 6 months,*

*You know that no painting or posting the walls*

*That is impossible, strictly forbidden.”*

#### Utilization of ITN

The poems called the risk community to utilize ITN. The students defined the practice of sleeping under ITN as an important weapon to fight malaria. They advised that ITN use should become a normal part of daily actions: adapted by everybody during every night, all seasons, and never missed whenever owned. Moreover, the students urged for giving priority to pregnant women and children. Illustration captured by a grade 5 student from the Limmu-Kossa said,

*“…Let’s use bed net at every night*

*Tie it carefully up when we are awake.*

*Why do we damage it? Rather we care for it*

*We use it as it keeps our health*

*We eliminate malaria by using it.*

*Give priority for infants and pregnant women.”*

*We use it always and stay healthy*

*When not enough for all, play your obligation*

In the poems, ITN was also presented as precious material to care for through regular washing, drying under the shade, stitching when torn, and never used for unintended purposes like rope, sacks, and wraps*.* Interestingly, the students also depicted their educational and supportive roles in the act of caring for ITN. A grade 7 male student from Non-Benja stated,

*“…Get up, arise, the educated children*

*It is your turn to teach your community*

*Tell them to sleep under the bed net*

*Not just sleeping under it, they should also care for it*

*We should observe the net and follow every day*

*We search it out and stitch whenever we see torn*

*Never forget to tie up and suspend after using it.*

*It must also wash at every three-month*

*We use soap for a wash and shade for drying.”*

#### Seek treatment and drug-use for malaria

By their poetic skills, the students encouraged their audiences to seek treatment for malaria when symptoms are recognized. Through previous quotations, two main messages were inherent in the poems regarding treatment: malaria should be treated at the nearest health facility, and the treatment seeking should be made early that is within 24 h of the onset of symptoms. Regarding drugs, given the malpractices presiding in the community, the poems urged the community to complete taking drugs as prescribed by the professionals. A grade 5 female student from the Shebe-Sombo said in a local language,

*“…Yoo fuudhannee fixnem haaluma nuu gooti*

*Abbaano fitti dhukkuba taanaandhukkubni maalgooti*

*Siluma abbaanofi dhukkubaaf dawaaofiti*

*Qoricha mana yaalaa nama keennameeni*

*Fayyadamani osoo hin hanbisin*

*Hidda isheeni kuuttu fayyaaof ieegnani.”*

This stanza literally indicated that it is possible to control malaria by taking care of ourselves, particularly adhering to drugs as prescribed by health workers. One keynote the poet underscored was that it is not a disease that matters rather it is reluctance among the people to early seek care or adhere to the prescribed drugs.

#### Social and behavior change towards malaria elimination

There were interesting messages in the poems that called the entire community and stakeholders to eliminate malaria through collective efforts and engagement. Several principles that defined the social change towards malaria elimination were inherent in the poems: have a sense of ownership of elimination tasks (e.g.: cleaning environment and management of mosquitoes), feel responsible (e.g.: considering oneself as having a role in the task), social cohesion (e.g.: building networks and keep unity to accomplish pertinent practices mentioned earlier), and collective efficacy and engagement (e.g.: acting together and involvement of all segments). A grade 8 male from Gera said the following:

*“We will eliminate malaria by working together and keeping unity*

*We will educate our community and take care of our acquaintances*

*Parents and children all contribute to eliminate malaria.*

*We will start working to eliminate by this year*

*So that it won’t kill us again like any time ever before*

*We will not pass malaria for the next generation*

*We will eliminate malaria by working hand in hand*

*Let teachers eliminate malaria so that the community develops*

*Let you teach and consult our farmers*

*Let’s do what we can do and work with health providers*

*Accept their advice and observe what they say.*

*Let's get up in groups and individuals to eliminate it from our country.”*

Another grade 5 student from the Gera district wrote:

*“…Let’s get up and make a campaign*

*To remove malaria from the country*

*Wakeup, especially the educated students*

*Let’s start teaching from the uneducated student*

*Let’s go to our people to eliminate malaria*

*We drain stagnant waters by working together*

*Rise and stop it by standing in unity*

*Be strong everyone, and protect yourself”*

Another *grade 8 male student from Bator-Tolay wrote:*

*“... Wakeup to eliminate malaria*

*Strengthen yourself and fasten your belt*

*Remove the ways of spreading the disease*

*Be united as working alone will make you tired*

*As elders saying bonded strings will tie an elephant (read 2X)”*

### Metaphors, similes, and personifications promoted malaria preventive practices

It is not uncommon for poets to use figurative languages. Details of the figurative speeches presented in a separate article. The current report was limited to few examples of metaphors, simile, and personifications.

#### Metaphors

Metaphors were expressions used by directly representing perceptions and practices about malaria according to mental models (symbols, objects, situations, etc.) of the local community. War and deaths were symbolic representations used to indicate the need to engage in the fight against malaria and its prognosis if the fight is handled reluctantly. A grade 7 student from the Nono-Benja said,

*“...It is not as some people think malaria is mild*

*It kills if you don’t go to the health facility*

*Let's get up for the fight don’t be lost in the battle*

*Oh, people of Jimma rise for the solution*

*Defeat the war against malaria.*

*Why are we beaten while we can win it?”*

The death itself was expressed as “being consumed or eaten” i.e. to imply that deaths from malaria are premature and preventable and should be modified through social and behavioral changes*.* An 8th grader female student from the Gera expressed,

*“..Malaria wants to take the lives of many people*

*Just like yesterday when it consumed the lives of my people...”*

In the poems, ITN was represented by a trap i.e. a mental picture used by students to draw the attention of their audience; rural people use ‘trap’ for hunting animals e.g.: pigs, monkeys, etc. that damage their farming. This indicated that there is a need to actively use ITN to trap mosquitoes. As the poorly maintained traps loss to catch animals that damage farming, improper use of ITN makes us lose the game over malaria. The mosquito is sketched as a life-stealing thief. A grade 6 male student from the Botor-Tolay said,

*“I will tie a trap and spend the night under it,*

*Where do you get me? Why do you try to bite me?*

*If you hang the trap, mosquito don’t bite you anymore*

*So, my people don’t joke regarding bed net,*

*Utilize it properly, don’t pierce and discard it*

*Hanging the trap on our bed, we will capture and trouble it*

*Finally, it will cry anxiously and left in there...”*

A grade 5 female student, from the Shebe-Sombo represented mosquito as a life-stealing thief:

*“…We know that malaria has many lives*

*Why do we forget to care for our life?*

*The Creator wants us to protect ourselves*

*Why do not we use the bed nets?*

*So that a thief will not enter the house*

*Through the door that we have opened (read 2X).”*

#### Similes

The similes are close to metaphors, but the liken malaria perception and practices were linked to something else instead of direct representations. Similes were mainly used to express distinguishable signs/symptoms. For example, fever was expressed like a sunny hot day, and chills in terms of rainy season weather. A grade 5 student from the Limmu-Kossa said,

*“…It makes me hot like the sun burning sun in the daytime*

*It also makes me shiver like rainy season…”*

#### Personifications

Personifications were mainly assigned to malaria and mosquitoes. The mosquito’s acts of biting and causing malaria disease assumed character of people who do evil things while looking good in wiles (e.g.: some people attack others while laughing). A grade 6 male student from *Botor-Tolay said,*

***“…****This mosquito has a lot of threatening activities*

*It has a sharp mouth to feed on human blood.*

*It moves from one person to other looking normal person.*

*It hurts a lot though it looks harmless when it comes to you.”*

Another grade 6 male from Gera expressed:

*“Biting is caused by a female mosquito.*

*It carried poison and attacked the community*

*It doesn’t fear God while harming people*

*It works very hard to replicate itself”*

Another *grade 8, female student from Botor-Tolay personified malaria as:*

*“…We have been suffered from malaria*

*It builds a house and reproduced in our body*

*…It finally eats everyone it caught.”*

## Discussion

The study explored 602 contents and organized into twenty-one categories under five central themes. The central themes were knowledge about malaria; risk and threat perceptions, Misconceptions and malpractices, the effectiveness of preventive methods, and calls to adaptations of practices. Also, metaphoric expressions and personifications were the commonest forms of figurative speeches used in the poems.

Causations and mode of transmission of malaria were one of the main contents raised across the poems. Knowledge of the correct cause of malaria i.e. the parasite Plasmodium transmitted through the bite of female anopheles mosquitoes was the commonly introduced content of the poems. Fever, chills, and headache were the commonest signs mentioned in the poems. ITNs and environmental cleaning were the main message contents transmitted towards increased knowledge of preventive actions. Myriads of malaria studies and behavior change-oriented programs emphasized similar knowledge contents [42–44]. Moreover, it is important to know the correct causation and prevention of malaria to enhance the adoption of preventive and treatment actions [45, 46].

The poems picked up misconceptions, beliefs, and malpractices presiding in the community. The dominant misconceptions were related to the causes of malaria. Some of the lists include food shortages, hungry, dirty foods, food items like sugar cane or maize, etc. Numerous studies reported similar misconceptions about causes [47, 48]. There were malpractices in the community regarding malaria mainly focused on preventive actions. These include misuse of ITN and sharing and interruption of drugs, similarly, pieces of studies revealed such acts are rampant in Africa [49–51]. Moreover, identification and reflection of misconceptions, beliefs, perceptions and malpractices affecting a given behavior is the central concept of sociocultural model [24, 25]. Therefore, this underscores that the students should have to work hard to exhaustively explore the behavioral factors and make the community to realize the actual behavior through SBCC.

Students in primary schools addressed content that enhanced the community appraisals of the threats from malaria. Some studies in Ethiopia indicated the incidence and perception of risk to malaria are significantly falling [4, 20, 52, 53]. Despite the falling trend, the poems presented the need to feel the risk and severity of malaria. Particularly, this study revealed that the severity of malaria was the most dominant content presented across the poems accounting for 101 (16.8%) of the entire specific messages. Premature deaths and wars were some of the metaphors used to initiate the feeling of risk and engage in practices.

Perceived effectiveness of preventive measures was one of the contents in this study which includes bed net, environmental modification, and spraying chemicals. Specifically, the effectiveness of sleeping under bed net was mostly included. This implies that communities’ perception of the effectiveness of ITN utilization was high. Finally, the utilization of ITN to prevent bites of anopheles mosquito as well as malaria would be increased. However, the study explored that message related to the vulnerability of malaria was very low. This implies that the community might be slightly motivated to act the malaria control measures. So, it is important to design messages that increase the perception of community members who remains susceptible to malaria. This is because; according to the EPPM model, it is recommended to design a message that increases perceptions of the community to make them remain susceptible to the diseases if they feel applying preventive measures is easy and effective but have little fear about the risk of the diseases [52–54].

Calls to adapt malaria prevention and control practices by the community and social changes were one of the major contents of the poems. Cleaning surrounding environments to destroy mosquito breeding sites (draining stagnant water and swampy areas, removing broken materials, and cutting the grasses and leaves of some plants) was a leading call. Use of chemicals, i.e. IRS was mentioned in the poems with precautionary (remove household utensils, ventilate houses, no plastering or painting walls until 6 months of spray, etc.) measures. The second most common call was sleeping under an ITN every night and season. Global strategies specify habitat modifications, ITN, and IRS as core physical and chemical mosquito control mechanisms [3, 5, 8, 43]. Though they were not dominant ones, seeking treatment for malaria symptoms and adhering to the prescribed drugs were conveyed in the poems. Likewise, ENMP indicates symptoms of malaria should be treated with appropriate drugs after confirmed testing, with 100% adherence to prescriptions within 24 h of the onset of fever [3, 43]. Fascinatingly, beyond calls to individual or household behavior changes, the poems sketched social changes for malaria control and elimination. The students raised critical values like the sense of ownership, feeling responsible, social cohesions, and collective engagement to eliminate malaria. Similarly, health communication interventions emphasize two forms (behavior and social) of change. The social changes were marked by indicators like community participation, engagement of local leaderships and networks, social cohesions, sense of ownership, sharing and feeling of responsibilities, etc. [11, 55].

Naturally, the poems are known in artistic literature contents i.e. use of figurative speeches. There are numerous types of figurative languages to convey messages in any artistic communication: some of them are metaphors, similes, personifications, hyperbole, litotes, irony, paradox, synecdoche, pun, etc. [26, 27, 33]. The investigators presented the details of the literature contents of the poems in a separate article. Nonetheless, two examples of metaphor (about ITN) and personification (about malaria mosquitoes) are used below. This is to explain how messages effectively conveyed in poems, creating contexts and mental models or heuristics for learning or change. Metaphorically, the poems represented ITN by traps that effectively catch harmful causative agents i.e. mosquitoes in this case. Such mental models could easily convince the public, particularly in rural settings, where traps are used to safeguard farming from animals that damage crops. Thus, audiences of the poems could visualize an active use of ITN as a mechanism of putting mosquitoes in a trap i.e. to prevent an attack. Moreover, in the poems personification was allotted to mosquitoes. It was given the characters of someone who do evil on human beings while resembling friends in their wiles. This personification can easily elaborate that mosquitoes live in the community, despised, and looked as safe, but, unexpectedly turn out to be harmful and killing. In doing so, the poems give warning to take care of malaria mosquitoes practicing malarial control measures. Evidence indicated that uses of local contexts and mental heuristics are effective ways of communicating perceptions, attitudes, and promoting practices [10, 56–58].

Overall, the poems developed by primary school students were rich in message contents and also promoted a wide range of malaria prevention and behavior change programs. For example, most of the contents in the poems were targeted by national strategies, RBM’s SBCC indicators, stated in malaria elimination framework, and global technical strategy of 2016–30 [5, 8, 11, 55]. To mention some, perceived susceptibility, severity, attitudes, self-efficacy, social norms, and practices related to ITN, IRS, environmental, and treatment were the dominant social and behavior change indicators of these programs. Interestingly, a study indicated that there were behavior changes observed in settings where these poems were developed, particularly in knowledge, ITN usage, and precautions concerning IRS [20]. Perhaps, the poems contributed to a social change. Moreover, the students developed the poems based on a balanced, fact-based, and truth-oriented malaria messages and conveyed the community perceptions, emotions, feelings, perceptions and practices [59]. Furthermore, dissemination of information through different internet-based social media is very important to easily and quickly communicate the message to the wider society. This is because; at the current time, the availability of internet access and the peak of a digital industrial revolution and new technological revolution and if students use it cautiously, it will help rapid information dissemination [60].

Therefore, engaging students in generating poems may provide contexts for change and predict malaria prevention and control actions in this era of elimination.

### Strength and limitation of the study

To the best of the investigator’s knowledge, there was limited published work in this area in Ethiopia. Therefore, limited literature was used to discuss the study findings. These poems were developed and disseminated by primary school students following a school-based malaria project aimed to advance community knowledge and practices. The contents could have been outputs of the knowledge they got from the project. This means students in schools that were not targeted by the project may not produce poems that are rich in malaria message contents in a similar manner. Thus, minimal support could be required to engage students in such effective agencies. Undeniably, primary school students were not professional and experienced poets. The content analysis presented in the current document may emphasize the connotative than hidden meanings. Nonetheless, there are still clues that the student’s skills can be nurtured, utilized, and promoted.

### Implications

The study found that the contents of poems developed and disseminated by primary school students were rich enough to create awareness and change the behavior of the local community towards malaria control and elimination program. Moreover, the project brought behavioral changes among the community members, especially on knowledge, ITN usage, and precautions concerning IRS [20]. This implies that involving school students in a community-based health programs, especially on the malaria control and elimination program is fundamental. Therefore, program planners, implementers or different stakeholders interested to implement such community-based intervention on similar context should engage school students who are positively competitive to develop messages that reflect the local norms, beliefs, values, perception and practices, and disseminate the messages to convince the local community to bring the desired or intended behavioral change. Moreover, the findings of this study can be used as an input for the program planners or partners who work on malaria-related projects. It can also be used as input and insights for researchers, especially in the discipline of health promotion, health education and health communication regarding school-based behavior change, and message development programs.

## Conclusions

The poetic content analysis indicated the poems developed and disseminated by primary school students were rich enough in message contents and also promoted a wide range of malaria prevention and behavior change programs. This implies that the students can be a considerable source of dominant social and behavior change-oriented malaria messages, particularly in resource-limited rural settings. Therefore, involving primary school students in public health and malaria programs would be an effective approach in promoting knowledge, risk perceptions, attitude, and practices particularly insecticide-treated net use, and treatment of fever.

Messages about knowledge of malaria prevention methods, perceived severity, and the practice of ITN use, and cleaning the environment were the commonest in students’ poems. Nonetheless, messages about the vulnerability and seeking treatment for fever may still be required in the current context of the declining trend in malaria incidence and era of elimination. The uses of poems would be advantageous for creating learning contexts by using local beliefs and mental models for conveying messages in a convincing manner and figures of speeches including metaphoric expressions.

## Supplementary Information

**Additional file 1: Supplementary file 1**: Code book manual for analyzing malaria poems message contents.

**Additional file 2: Fig. 2**: Networks of themes and categories of message contents across the poems, Jimma zone, Ethiopia 2020.

## Data Availability

The datasets used and/or analysed during the current study available from the corresponding author on reasonable request.

## Abbreviations

AIM: Action and Investment to Defeat Malaria
GTS: Global Technical Strategy for malaria
IRS: Indoor Residual Spraying
ITN: Insecticide-Treated Net
ENMCP: Ethiopian National Malaria Control Programme
SBCC: Social and Behavioral Change Communication
PMI: President’s Malaria Initiative
RBM: Roll Back Malaria
WHO: World Health Organization
