# Supplementary material for: Primary school students’ poetic malaria messages from Jimma zone, Oromia, Ethiopia: a qualitative content analysis
Source: BMC Public Health. 2021 Sep 16;21:1688. doi: 10.1186/s12889-021-11641-8 (PMC8447558; doi:10.1186/s12889-021-11641-8)
Supplement: Supplementary file 1 — Additional file 1: Supplementary file 1: Code book manual for analyzing malaria poems message contents. [file 12889_2021_11641_MOESM1_ESM.docx]

**Supplementary file 1: Code book manual for analyzing malaria poems message contents**

| Codes | Definitions | Examples |
| --- | --- | --- |
| Knowledge about malaria and its prevention | Description of information about malaria and its prevention measures in the poetic message of primary school students. It includes description of causes and modes of transmissions, mosquito breeding and biting behaviors, sign and symptoms, and ways of prevention, care of ITN, treatment and control of malaria. | *“The causes of malaria is plasmodium parasite and transmitted by female anopheles mosquito and there are ways of prevention of malaria like that of hanging bed net every day, spraying chemicals in the house and cleaning the environment “.* |
| Knowledge about sign and symptoms of malaria | Description of distinguishable signs and symptoms of malaria which ranged from feeling hot, headache, feeling cold, weakness, joint pain, relapse, vomiting, and etc. | *“These are the symptoms of malaria: it warms the body, causes headaches, makes you vomit and hates food, weakening the body, once you get better, it comes again and again, for infants and pregnant mothers by causing to feel cold it shakes the body, and it throws their head down.”* |
| Knowledge about ways of prevention and control | Contents that describe a means of preventing mosquito bites and malaria including sleeping under ITN, spraying house with IRS, disposing broken material that hold water, avoiding stagnant water, avoiding mosquito breeding site and keeping clean the environment | *“For this (malaria) diseases, it has many ways of prevention methods: cleaning effectively/correctly breeding site of mosquito, don’t left the toilet open, avoiding broken materials that hold water, disposing and draining ponds that holds water, removing dead waste from the environment,… hanging bed net by giving priority to sleep under bed net for pregnant women and under five children If there is shortage of bed net, because they are easily attacked and have weak body…. spraying chemicals in the house”.* |
| Knowledge about causation and mode of transmission of malaria | Contents that describe about how malaria diseases are happen /occurred. It includes that causation of malaria by plasmodium species and bite of anopheles mosquito when previous bite was made with someone who has the parasite plasmodium in their blood stream. | *“The way it (malaria) is transmitted is by female anopheles mosquito and the parasite is called plasmodium. It is transmit by sucking of one person’s blood which contains plasmodium parasite) to another person. Further it transmits the diseases for the peoples.”* |
| Knowledge about mosquito biting behavior | Description of contents about biting behavior of mosquito i.e. biting is mainly performed at night and morning time, feature of biting and feeding habits in by their poems messages. | *“Mosquito has a very wide mouth when it bites a person enters a person’s bloodstream and its bite is for the sake of reproduction and time of biting is during night and morning time.”* |
| Knowledge about mosquito breeding behavior | Contents that describe suitable breeding sites of mosquito like that of stagnant water, broken materials that hold water, grass, and swampy area in the students’ poetic messages. | *“It (mosquito) breeds on broken materials that hold water, stagnant water and grass (16, years old, grade 8, and male student in Limmu-Kosa district) …. And during winter and summer, it assembled and breeds on stagnant water and lays its eggs 50-70 at a time and completes its life cycle of metamorphosis. Then there will be plenty of them all over the world”.* |
| Knowledge about ITN care | Description of how ITN is cared in by their poems messages including washing of ITN with soap within 3 month and drying under shadow stitched when torn, and never used for any other purposes. | “*If ITN becomes dirty, wash with soap and dry it under shade and if it gets tear, repairing or mending it. And also bring up at 12 O’clock am LT…teach the people to not use for other purpose.”* |
| Misconception ,belief and malpractice in the community | Contents that describe presence of misconception, beliefs, and malpractice regarding malaria and its prevention and control methods, in by their poems’ messages and should be improved. | *“There are beliefs of boiling coffee as a treatment for malaria and using of previously saved drugs over the counter and using of bed net for tying cattle and laying grains in our community.”* |
| Misconception about causes | Description of contents in by their poems that is wrongly believed causes of malaria in their community like that of hungry, sleeping outdoors ,eating sugarcane, dirty foods, cold weather and etc. perceived as a causes of malaria. | “*Every person’s says where is the source (causes) of malaria… the diseases is not caused by lack/shortage of food, and sleeping outdoors”* |
| preventive and treatment malpractice | Contents that describe people believe in and follow local preventive/treatment modalities which need improvement. It includes malpractices concerning treatments like using herbs, and previously saved drugs, buying drugs over the counter, sharing from each other and also using bed net for laying grains, tying cattle and toilet construction. | *“Why drug interruption becomes our behavior? Isn't it true that we spend money to be saved? And also there is giving of previously used and saved drugs for other persons… also there is discarding of the drugs which got from health facility. Please utilize it appropriately in order to become a medicine (treatment)”* |
| Threat perception from malaria and risk condition | It refers to contents that describe risk of experiencing and severity or seriousness of malaria.it includes risk conditions, perception of risk, and severity. | *“Malaria affects and killed all my people in the past and caused to decreased Production and Productivity of the farm. Then it exposed to them for starvation.”* |
| Perceived risk or vulnerability | Contents that describe perception of likelihood of experiencing malaria in by their poems messages which includes initiating the community to feel risk of infection by malaria or it serious form, and also indicating pregnant women and under-five children are at high risk of experiencing worsening forms of malaria due to their low immunity. | *“Malaria attacked all persons without permission and difference in age (children and aged persons)… every/all person are attacked or affected by malarial diseases it doesn’t fear the fatty persons and undermine the thin/slim persons it affects all persons”.* |
| Risk condition | Description of presence of risk condition that exposed to biting of mosquito and malaria in by their poems messages. It includes the presence of wastes, grasses, flat leaves of plants, swampy areas, stagnant waters, and broken utensils that are favorable for breeding of mosquitoes. | *“We stored water in the surface then it (mosquito) rests and stores the diseases on it. After that by biting it put the diseases on us. “Why do people stop pouring stagnant water in our area? .... Due to we didn’t Clean our environment and disposing stagnant water .It breeds there then it bites us.”* |
| Perceived severity | Contents that describe perception about seriousness of malaria that happened in community. It includes description of causing loss of human life, economical loss, damage or limit to a person’s physical or mental abilities, and its effect on farming activities in by their poems messages. | *“Malaria is very heavy diseases by spreading the diseases bothered everyone. It kills and disabled the persons quickly without assuming or recognizing the presence of the diseases…and also it doesn’t says father, mother, female and children it bans/destroy from the earth and cause to sorrow/cry every ones”.* |
| Perceived effectiveness of preventive measure | Description of contents that describes ability of preventive measure in helping the community to keep from malaria. It includes perception of effectiveness of bed net, IRS, cleaning the environment, treatment and drugs. | *“Using bed net every night, cleaning environment and spraying chemicals in house are effectives in protecting bites of mosquito and malaria.”* |
| Effectiveness of ITN | Description of perception about effectiveness of ITN in keeping bite of mosquito and malaria. It includes that active use of ITN can safeguard a family and community from risk of malaria and it can trap malaria causing mosquitoes. Considered as a preventive material in the fights against malaria. | “*You can protect yourself from malaria by sleeping under bed net …I will suspend and sleep under bed net. Where you get me. Why do you bother to bite me? If you tauten bed net, it didn’t get to bite for the second time”.* |
| Effectiveness of IRS | Contents that describe perception about working of spraying IRS in house and on water bodies in peak breeding seasons to kill mosquitoes that causes malaria in by their poems messages. | *“It is possible to eliminate/prevent malaria disease, by spraying antimalarial chemical in the home.”* |
| Effectiveness of cleaning the surrounding environment | Description of perception about regular cleaning of compounds and surrounding environments as effective means of reducing risk conditions and chances of mosquito breeding. | *By looking (cleaning) our environment hourly and destroying stored water in on time and by checking our villages’ sanitation regularly, we will far away the mosquito from our villages….We can prevent ourselves from malarial diseases, by keeping our environment clean”.* |
| Effectiveness of seeking at health facility | Contents that describe nearest health facility as effective ways of testing, ruling-out and managing malaria.. | *“If you infected with malaria, by going to the nearest (local) health facility, you will get a treatment of malaria… If you see the symptoms of malaria, we should not die by sleeping in bed, but immediately by getting treatment saving life is a public duty”.* |
| Effectiveness of drugs | Description of contents that describe effectiveness of drugs in protecting deterring or avoiding of malaria. It includes that failing to complete anti-malaria drugs result in relapse, and professional prescriptions as the only best means of taking drugs. | *“If you look symptoms of malaria, take the persons to health facility, why do malaria eat us by knowing this (knowing of health facility as the best place of getting of treatment ).Finishing a drugs which we were given by health provider is a must for us. It is our fault that leaving unfinished but if we finished the drugs, it becomes disappear from our body”* |
| Call to adopt malaria control practice and social and behavioral changes | Description of contents which explicitly called the exposed community and any other listeners/readers to adapt the practice of cleaning their surrounding environment, ITNs use, IRS, seeking treatment when symptoms are present and appropriate drug-use and social change and collective engagement on eliminating malaria from their community | *“We will eliminate malaria by working together and keeping unity. We will educate our community and take care of our acquaintances. Starting from father to son contribute for elimination of malaria. We’ll eliminate malaria from beginning this year. It did not kill us for the second time. We will not pass it on to the next generation. We will eliminate malaria by planning and working together or supporting each other (by connecting with hand by hand).”* |
| Clean the surrounding environment | Contents that describe performing of cleaning the environment to achieve the control over mosquito and malaria. It includes managing small water bodies/swampy or any surrounding conditions suitable for breeding including broken utensils, leaves of some plants like incent, and other wastes. Accordingly, draining waters, filing swampy areas, cutting leaves, and burning of wastes suggested as actions in the poem of students. | “*It (mosquito) breeds on broken materials that hold water, in stagnant water, and also in abundant in grass. So, if we get broken material, we will dig for it and buried in a pit (hole). For varies waste, it will be burned. Sleeping water will be avoided. Grass will be removed. And the environment will be checked for the sanitation regularly”* |
| Take precautionary measures for IRS | Contents of description that commanded the community to adapt IRS. It includes precautionary measures that prevent unintended effects of IRS by closing house for few minutes after spray and properly ventilating for hours. | *“Spraying anti-malarial spray is the third one (prevention methods).Let’s get out after spraying it. We will never enter in to home up to two hour of spraying and we will not open the door up to 15 minutes.”* |
| Utilization of ITN | Description of contents that calling the exposed community to actively engaging in utilizing ITN every night, by giving priority to pregnant women and under five children. It includes calling of the community to use ITN as a normal part of daily action and adopt everybody to every night and at all seasons and at the time of limits to give priority for pregnant women and under five children never missed when they owned and caring of ITN by regular washing; drying under the shade, and stitching when torn. | *“We use bed net every night and also we will tie bed net and spend the night under it, where you get me? Why do you bother to bite me? After two month of owned bed net, we used for a rope, for tying calf; by forgetting our health we used it for cattle. These are not right way and why are we foolish. So, when we get the bed net we will use carefully in save. Why do we damage it? We will keep our health and eradicate malaria by using it. We'll tie the bed net and stay our healthy…if we are in trouble of bed net access, prioritizing infants and pregnant women are our obligation.”* |
| Seeking treatment and drug use for malaria | Contents that encouraging the audiences to seek treatment when distinguishable sign are present.it includes treating of malaria at health facility other local practices may not work and seeking treatment within 24 hours of onset Following seeking of treatment is appropriate use of drugs meaning practicing professionals’ prescriptions at any expense by completing the drugs according to the scheduled time of taking. | *“What will it (malaria) do, if we take completely the drugs? If the person is a disease on his own. What to do malaria? First of all, the man is a treatment for his own diseases. So, a man who destroys his /her drugs will sorrow by downing his head… finishing the drugs which was given by health providers is a must for us. Leaving unfinished is our fault. If we take the drugs completely the diseases will disappear from our body.”* |
| Social change towards malaria elimination | Description of contents that calling the entire community and stakeholders to eliminate malaria through collective efforts and engagement. It includes practice of encouraging the need to have sense of ownership of the task of eliminating malaria (cleaning environment and management of mosquitoes), feeling responsibility (considering oneself as having role in the task), social cohesion ( building networks and keep unity to accomplish pertinent practices mentioned earlier), and collective efficacy and engagement (acting together and involvement of all segments) | *“Let's get up and make a campaign to leave malaria from the country. Especially educated students, let's get up and teach uneducated student about malaria. And also come on my people, let's remove and eliminate malaria by disposing stagnant water and understanding each other’s and also by raising or standing in unity. Everyone knows and protect yourself from the diseases (malaria).”* |
| Metaphors, similes, and personifications promoted malaria preventive practices | Contents that describes different figurative languages in order to convey beliefs, norms and practices related to malaria .It includes metaphors, similes and personification, irony ,litotes, metonymy, paradox, pun, synecdoche, and paradox. | *““If we expose ourselves to the diseases, who will fight us? If the person becomes diseases on his own, what malaria to do? A thief will enter the house through a door that he/she has opened. This mosquito has a lot of threating activities or works to do but when you see it seems like flies. It has a very wide mouth and feeds on human blood. It moves from person to person to lead its life.”* |
| Metaphors | Contents that describes an expressions that are used to directly represent perceptions and practices according to mental models of the local community including proverbs, symbols, objects, situations, and phenomena. | *A person looks malarial diseases as a bit diseases or lightest disease but if you don’t go to health facility, it kills the persons… Let's get up and protect ourselves, don’t lost human life While there is a solution. “Particularly, the people of Jimma, let’s prevent (defeat) war of malaria. Why does it beat us while we can win it?”* |
| Similes | Description of contents in by their poems to express malaria and its relating things with a connective such as “like”. It includes an expression of anopheles mosquito, malaria and sign and symptoms of malaria related to another thing with a connective “like”. | *“It (malaria) makes me fevered like that of a strong sunny season… it makes me a colder and shivers like that of a heavy rainy season… The name of mosquito is anopheles mosquito… when you see (anopheles mosquito), it seems like flies.”* |
| personifications | Description of contents in the poems messages by using or assigning human characters to describe or explain situations or inhuman objects in relation to causes, signs, prevention and treatment of malaria. | *“From mosquito of malaria, biting is caused by female mosquito. It carries poison and finished the community. It doesn’t fear the creator (God). And also in order to bans and sorrow/cry the people it works very hardly and replicates itself overly.”* |
