# Supplementary material for: Primary school students’ poetic malaria messages from Jimma zone, Oromia, Ethiopia: a qualitative content analysis
Source: BMC Public Health. 2021 Sep 16;21:1688. doi: 10.1186/s12889-021-11641-8 (PMC8447558; doi:10.1186/s12889-021-11641-8)
Supplement: Supplementary file 2 — Additional file 2: Fig. 2: Networks of themes and categories of message contents across the poems, Jimma zone, Ethiopia 2020. [file 12889_2021_11641_MOESM2_ESM.docx]

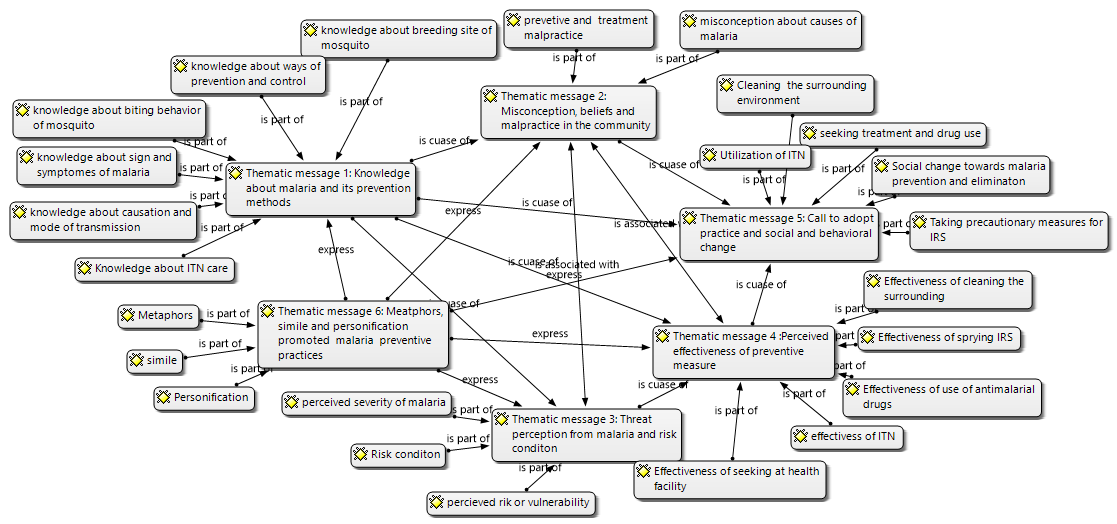


Fig. 2: Networks of themes and categories of message contents across the poems, Jimma zone, Ethiopia 2020
